# Modulation of Endothelial Function by TMAO, a Gut Microbiota-Derived Metabolite

**DOI:** 10.3390/ijms24065806

**Published:** 2023-03-18

**Authors:** Giulia Querio, Susanna Antoniotti, Federica Geddo, Renzo Levi, Maria Pia Gallo

**Affiliations:** Department of Life Sciences and Systems Biology, University of Torino, Via Accademia Albertina 13, 10123 Torino, Italy

**Keywords:** endothelium, vascular function, angiocrine factors, TMAO, gut microbiota

## Abstract

Endothelial function is essential in the maintenance of systemic homeostasis, whose modulation strictly depends on the proper activity of tissue-specific angiocrine factors on the physiopathological mechanisms acting at both single and multi-organ levels. Several angiocrine factors take part in the vascular function itself by modulating vascular tone, inflammatory response, and thrombotic state. Recent evidence has outlined a strong relationship between endothelial factors and gut microbiota-derived molecules. In particular, the direct involvement of trimethylamine N-oxide (TMAO) in the development of endothelial dysfunction and its derived pathological outcomes, such as atherosclerosis, has come to light. Indeed, the role of TMAO in the modulation of factors strictly related to the development of endothelial dysfunction, such as nitric oxide, adhesion molecules (ICAM-1, VCAM-1, and selectins), and IL-6, has been widely accepted. The aim of this review is to present the latest studies that describe a direct role of TMAO in the modulation of angiocrine factors primarily involved in the development of vascular pathologies.

## 1. Endothelial Dysfunction

Endothelial cells (ECs) line the blood vessels and have multiple functions that are not only linked to nutrients and oxygen supply to peripheral tissues [1,2]. Indeed, ECs are involved in the regulation of vascular homeostasis and tissue regeneration through the secretion of paracrine factors named angiocrine factors [2,3]: cytokines, chemokines, and growth factors released by ECs show how these cells play a crucial role in endothelial function and dysfunction [1].

**Figure 1:**
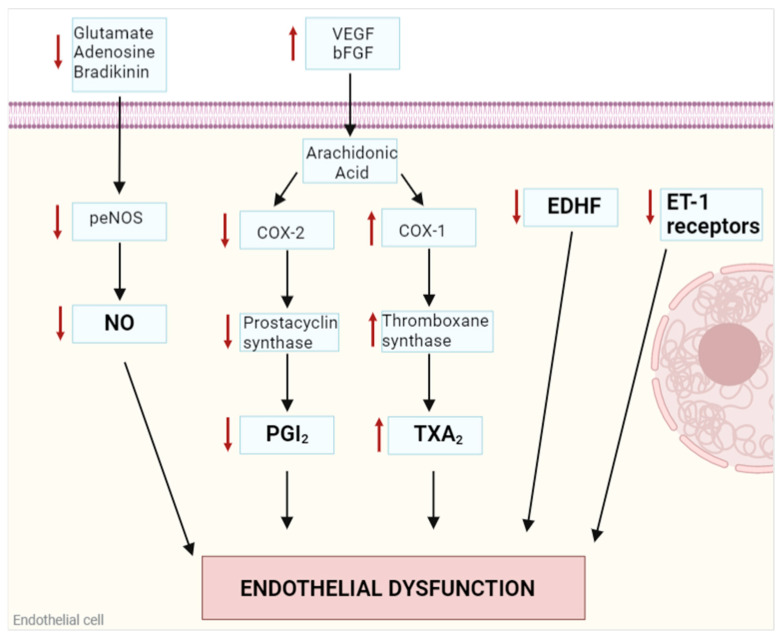
Endothelial dysfunction is regulated by different autocrine and paracrine factors released by endothelial cells. Different mediators are involved in the impairment of the vasoconstriction/vasodilation modulation: inhibition of eNOS through its dephosphorylation at Ser1177, which reduces NO release from ECs; downregulation of COX-2 and prostacyclin synthase, which are involved in PGI_2_ synthesis from arachidonic acid; upregulation of COX-1 and thromboxane synthase, which increase TXA_2_; and a decrease in EDHF release and reduction in ET-1 receptors on EC membranes are all factors that contribute to the contraction of the vessel walls. eNOS: endothelial nitric oxide synthase; NO: nitric oxide; EC: endothelial cells; VEGF: vascular endothelial growth factor; bFGF: basic fibroblast growth factor; COX: cyclooxygenase; PGI_2_: prostacyclin; TXA_2_: thromboxane A_2_; EDHF: endothelium-derived hyperpolarizing factor; ET-1: endothelin-1. Image created with Biorender.com.

Endothelial dysfunction is a pathological condition characterized by an improper balance between vasodilatory and vasoconstrictory mechanisms and it is considered the hallmark for atherosclerotic and other cardiovascular diseases burden [4,5] (Figure 1). The principal actor in this condition is nitric oxide (NO), a gasotransmitter synthesized by endothelial nitric oxide synthase (eNOS), implicated in the relaxation of the smooth muscle cells of the vessel wall. Activation of eNOS through its phosphorylation at Ser1177 [6,7] is regulated by shear stress, probably through a flow-induced release of acetylcholine [8] or by other circulating molecules such as bradykinin [9], adenosine [10], and glutamate [11]. Inactivation of eNOS through its dephosphorylation at Ser1177 and phosphorylation at Ser116 induces a strong reduction in NO release with subsequent vasoconstriction [6,7]. An improper balance in eNOS activation/inactivation can cause NO decrease and vascular contraction, leading to endothelial dysfunction [4].

Among other endothelium-mediated vasodilatory factors, prostacyclin (PGI_2_) is synthesized from arachidonic acid after vascular endothelial growth factor (VEGF) or basic fibroblast growth factor (bFGF) stimulation through a linked reaction between cyclooxygenase (COX)-2 enzymes and prostacyclin synthase. PGI_2_ modulates the vascular tone in a complementary way to NO [4,12,13]. Endothelium-derived hyperpolarizing factor (EDHF) is now identified with a complex intercellular pathway, starting from an intracellular Ca^2+^ increase in ECs and following with the efflux of K^+^ through Ca^2+^-sensitive K^+^ channels. The consequent extracellular increase in K^+^ concentrations induces the efflux of K^+^ from vascular smooth muscle cells through Na^+^/K^+^ ATPase and inwardly rectifying K^+^ channels, resulting in membrane hyperpolarization, a decrease in intracellular Ca^2+^, and vasorelaxation [12,14,15]. Moreover, endothelial hyperpolarization can also propagate from endothelial cells to vascular smooth muscle cells via myoendothelial gap junctions [15].

Vasoconstriction is also modulated by specific EC-released factors as well, in particular, thromboxane A_2_ (TXA_2_), which is synthesized from arachidonic acid by the linked reaction of COX-1 and thromboxane synthase. TXA_2_ binds its receptor on the membrane of vascular smooth muscle cells and enhances their contractile tone through an increase in intracellular Ca^2+^ [12]. Endothelin-1 (ET-1) figures among the major regulators of vasoconstriction, acting both in paracrine and autocrine stimulation. As a paracrine factor, it binds to its specific membrane receptors on vascular smooth muscle cells, activating the inositol trisphosphate (IP_3_)-dependent Ca^2+^ release from intracellular stores and thus promoting contractility. In the autocrine way, ET-1 acts on ECs inducing the release of NO and PGI_2_. It has been discovered that when endothelial dysfunction is developing, a downregulation of ET-1 receptors occurs on EC membranes, thus favoring its binding to smooth muscle cells receptors and activating the vasoconstriction pathway [12,16].

Endothelial dysfunction is also strictly associated with an increased inflammatory response in ECs [17,18]. When inflammation occurs in ECs, an activated state in these cells starts a vicious cycle in which an increase in inflammatory cytokines and reactive oxygen species (ROS), and an imbalance between vasodilator and vasoconstrictor mediators influence each other, exacerbating the pathological outcome. Chronic inflammation due to exogenous insults or existing pathological conditions is directly involved in the increase in nuclear factor kappa-light-chain-enhancer of activated B cells (NF-kB) signaling [19,20,21]. First, NF-kB upregulation in ECs influences the activity of different enzymes involved in the release of vasomodulatory factors; for example, it causes reduced eNOS expression and activation, thus decreasing NO release [22,23]. Furthermore, NF-kB induces the upregulation of several angiocrine factors, such as interleukin(IL)-6 and intercellular adhesion molecule 1 (ICAM-1), that are strictly related to an endothelial dysfunction outcome and atherosclerosis development [24]. Among different angiocrine factors activated by NF-kB and involved in a positive feedback loop with ROS as previously mentioned [17,18], vascular cell adhesion molecule 1 (VCAM-1) [25,26], ICAM-1 [26,27], selectins, and cytokines are primarily involved in the development of endothelial dysfunction by starting the recruitment of inflammatory cells, like monocytes, neutrophils, leukocytes, and macrophages, and exacerbating the inflammatory response [20].

**Figure 2:**
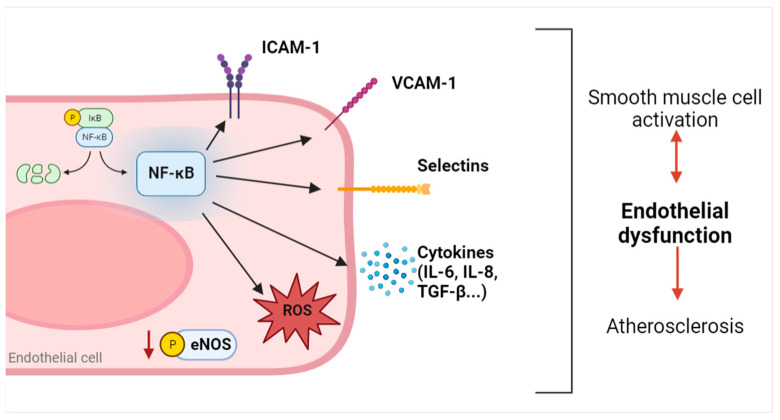
Schematic overview of the NF-kB pathway and angiocrine factors activated in endothelial dysfunction. NF-kB activation induces the upregulation of pro-inflammatory factors (ICAM-1, VCAM-1, selectins, and cytokines) and an increase in intracellular ROS. All these angiocrine factors, in combination with reduced eNOS phosphorylation, contribute to the exacerbation of endothelial dysfunction and smooth muscle cell activation, enhancing the atherosclerotic burden. ICAM-1: intracellular adhesion molecule 1; VCAM-1: vascular cell adhesion molecule 1; IL: interleukin; TGF-β: transforming growth factor β; ROS: reactive oxygen species; eNOS: endothelial nitric oxide synthase. Image created with Biorender.com.

NF-kB activation and the derived release of inflammatory cytokines also induce vascular smooth muscle cells activation, promoting further endothelial dysfunction and atherosclerosis [20] (Figure 2).

All the described mechanisms are primarily involved in the development and exacerbation of endothelial dysfunction. Different causes can contribute to this pathological condition [28]. Among all, genetic factors play a crucial role in the predisposition to all cellular responses involved in endothelial dysfunction, but environmental factors play an equally important role in destabilizing vascular homeostasis. In particular, diet and gut microbiota have been underlined to be among the most relevant environmental risk factors of endothelial dysfunction [29,30,31]. Recent studies show how modulation of dietary habits can improve vascular dysfunction, in particular in patients at middle and high risk of developing cardiovascular events [32]. Yubero-Serrano and colleagues analyzed through the CORDIOPREV (CORonary Diet Intervention with Olive oil and cardiovascular PREVention) study how diet composition is important in influencing the development of endothelial dysfunction. In particular, it has been widely accepted that the Mediterranean diet, in which almost 50% of the energy intake derives from carbohydrates, mainly complex, 35% from fat, mainly polyunsaturated, and 15% from proteins, mainly of vegetable origin, figures as the best nutritional approach to improve vascular health [32,33]. Indeed, the Mediterranean diet and other dietary approaches rich in vegetables, fruit, lean fish, and poultry prevent endothelial damage and reduce circulating levels of inflammatory markers; in particular, ICAM-1, VCAM-1, IL-6, and IL-8 expression is significantly lower in a diet poor in high-fats dairy products and red meat [33,34]. A relevant caution against red meat and animal-derived foods has developed because of their strong relationship with atherosclerosis. This correlation was initially explained through the high saturated fat content of these food matrices, while now the involvement of the gut microbiota as a key point connecting animal-derived food and atherosclerosis is also widely recognized [35]. Together with diet, aerobic exercise has been shown to be one of the best lifestyle interventions to counteract endothelial dysfunction and promote the colonization of a healthy microbiota in the gut, especially in old age, where multiple cardiovascular risk factors coexist [36]. Indeed, the gut microbiota synthesizes a plethora of metabolites from dietary sources that could be beneficial or detrimental according to the host status, and the composition of the gut microbiota could be influenced by endurance exercise [37,38,39,40,41]; alterations in the microbiota diversity, named dysbiosis, is associated with metabolic disorders onset due to an imbalance in released metabolites. Among all, L-carnitine, choline, and other amine-containing molecules, and not fats, present in animal-derived foods can be metabolized by the gut microbiota to form trimethylamine, which is absorbed in the colon and then oxidized by the liver to trimethylamine N-oxide (TMAO), whose role in cardiovascular health is widely discussed as its involvement as a direct cause or a marker of pathology remains unclear [35,42].

The aim of this brief narrative review is to summarize current knowledge on TMAO, particularly focusing on its role in modulating the release of angiocrine factors, directly taking part in the regulation of vascular homeostasis. The presented works were selected from PubMed and the keywords used for the search were: “TMAO OR trimethylamine N-oxide AND endothelial dysfunction”, “TMAO OR trimethylamine N-oxide AND angiocrine factors”, “TMAO OR trimethylamine N-oxide AND atherosclerosis”.

## 2. Trimethylamine N-Oxide (TMAO)

### 2.1. Sources

TMAO is a diet-derived compound that can be introduced directly through fish products or can be endogenously synthesized from dietary quaternary amine precursors, primarily L-carnitine, choline, betaine, and ergothioneine [43]. These precursors, particularly abundant in animal-derived foods, with the exception of ergothioneine, present in mushrooms, are metabolized by the gut microbiota to form the volatile molecule trimethylamine (TMA). Crucial genes expressed by the gut microbiota have been implicated in the synthesis of TMA, a critical process for the endogenous synthesis of TMAO, and thus for its accumulation in the host: choline-TMA-lyase and its activating protein (*CutC* and *CutD*), the Rieske-type oxygenase/reductase (*CntA/B*), and the L-carnitine-TMA-lyase (*YeaW/X)* [44]. Different bacterial strains express these cluster genes, comprehensively described elsewhere [44]. Alteration of the gut microorganism milieu can influence the synthesis of a plethora of secondary metabolites that are directly involved in pathological onsets. Indeed, it is now widely accepted that the microbial diversity of the gut is strictly influenced by the diet and vice versa, as the gut microbiota’s metabolism of different diet-derived compounds can influence the host’s health [45]. In particular, in addition to TMAO, several other gut microbiota-derived metabolites have been implicated in the enhancement of cardiovascular disease mortality, such as secondary bile acids and tryptophan and indole derivatives, as comprehensively described by Sanchez-Gimenez and collaborators [46].

Once TMA is synthesized, it reaches the liver through portal circulation, where the flavin-containing monooxygenase 3 (FMO3) catalyzes, first with a reductive half-reaction and then with a successive oxidative half-reaction, the formation of TMAO [47]. While studies in the past considered the liver as the principal tissue expressing the FMO3 isoform, new evidence suggests different sites where the enzyme is particularly activated. Indeed, its expression has been also outlined in aortic tissue [48], adrenals, and lungs [49], and these tissues could extend the availability of TMAO synthesized from TMA. Once formed, TMAO is released from the liver to the systemic circulation by specific transporters, as described in several studies. In particular, the ATP-Binding Cassette (ABC) transporters, ABCB1, ABCG2, ABCC2, and ABCC4, mediate the efflux of TMAO from hepatocytes [50]. Some TMAO reaches peripheral organs through the circulatory system and some is excreted through urine. Its entry into renal cells is mediated by organic cation transporter 2 (OCT2), while its output is mediated by the same transporters expressed in the liver [50,51,52].

### 2.2. Biological Functions

The first studies regarding TMAO are linked to its properties as an osmolyte. Indeed, higher concentrations are present in cartilaginous fishes and Osteichthyes that live in deep water. In these organisms, TMAO’s functions are primarily related to stabilizing protein structure against the high osmotic and hydrostatic pressures that characterize certain environments [53]. In particular, cartilaginous fishes also have high levels of urea that has the function of balancing the osmolarity of extracellular fluids, but which could be detrimental to protein stability if not properly balanced by other osmolytes, such as TMAO [47,54].

TMAO activity as a protein stabilizer, favoring the native conformation over the denatured protein, has been widely studied and different models have been proposed. In the first described mechanism, high concentrations of TMAO seem to not interfere with the protein backbone, favoring a compact, native structure of the molecule [54,55]. In a further proposed model, TMAO figures as a stabilizer by binding to the protein side chains through hydrogen bonding [56]. The third model presents TMAO as a surfactant when localized in proximity to the folded protein [57,58]. Nowadays, it is not obvious which of these models acts first in stabilizing the protein structure because various factors, such as pH, can affect the protonated and deprotonated forms of TMAO, but what is certain is that its role is to balance the denaturing effect of urea [54].

Moreover, as TMAO is primarily synthesized by an enzymatic reaction in the liver, it may be reasonable to think of it as a detoxification product of its precursor, TMA. Indeed, FMO3 enzymes, the main enzymes involved in TMAO synthesis, have a primary role in the oxidation of drugs and xenobiotics to favor their excretion [47]. To support this hypothesis, different lines of investigation point out that high levels of plasmatic TMA can be related to some pathological outcomes. Indeed, improper function of FMO3 induces trimethylaminuria, or “fishy-odor syndrome”, which is characterized by an unpleasant smell from sweat, breath, urine, and body secretions. Severe pathological outcomes in patients presenting this syndrome have not been underlined, but, as is understandable, they all show psychological discomfort due to their smell, which inevitably causes shame and social distancing [59,60]. Furthermore, recent evidence underscores that TMA, rather than TMAO, shows detrimental effects in the cardiovascular and renal systems. In particular, TMA is cytotoxic for cardiac cells, acts as a perturbing factor for protein structure in animal studies [61], and it seems to be involved in the cardiorenal syndrome, favoring increased blood pressure, proteinuria, and glycosuria [62].

### 2.3. TMAO and Atherosclerotic Risk

Emerging evidence highlights a controversial role of TMAO in the development of cardiovascular diseases [63,64]. A first group of investigations points out a strong relationship between high plasma concentrations of TMAO and the development of a pathological state, depicting the molecule as a causal factor of cardiovascular diseases [65]. Indeed, Wang et al. found a dose-dependent association between TMAO-supplemented diets, pro-atherogenic macrophage phenotype, and atherosclerotic risk [66]. Furthermore, significant differences among patients with mild, moderate, or severe coronary atherosclerosis and TMAO plasma concentrations have been shown, with strong evidence correlating high circulating levels of TMAO and atherosclerotic burden [67]. Haghikia and colleagues showed a positive correlation between high plasma concentrations of TMAO and increased cardiovascular risk. Moreover, in this prospective cohort study, the authors outlined a strict relationship between increasing plasma TMAO concentrations and proinflammatory intermediate CD14^++^CD16^+^ monocytes [68]. Finally, Brunt and collaborators demonstrated a key role of TMAO in the induction of age-related endothelial dysfunction through the enhancement of oxidative stress and a reduction in NO release [69]. On the other hand, part of the recent literature has outlined a neutral role of TMAO, presenting the molecule as a marker, rather than a direct cause, of atherosclerosis. Indeed, even if TMAO plasma concentrations can be influenced by the diet, it has been accepted that high circulating levels of the molecule do not correlate with atherosclerotic extent [70]. Jomard and collaborators also showed that patients subjected to Roux-en-Y gastric bypass (RYGB), despite high levels of plasmatic TMAO, have improved vascular function and gluco-lipid profile, as expected after surgery, suggesting a neutral role of the molecule in the development of atherosclerosis [71].

These controversial results show how the role of TMAO in the onset of cardiovascular disease is still unclear. Certainly, plasma TMAO ranges need to be defined in order to understand its role as cause or effect in human pathology [72]. The following paragraphs explore, by discussing both in vitro and in vivo studies, some of the mechanisms ascribed to TMAO in the regulation of angiocrine factors involved in vascular homeostasis.

## 3. TMAO-Mediated Angiocrine Factors Modulation

As highlighted in previous paragraphs, TMAO seems to be directly involved in the pathological development of atherosclerosis. This section presents current knowledge on how TMAO can be considered the trigger of endothelial dysfunction through the direct modulation of the expression and release of some crucial angiocrine factors. All the data illustrated are also summarized in Table 1.

### 3.1. Nitric Oxide

Endothelial dysfunction is characterized by the impaired release of NO. As previously described, the gasotransmitter is involved in the regulation of vascular tone and its reduced bioavailability is one of the first manifestations of vascular disorders.

Moreover, in 1995, De Caterina and collaborators characterized a further regulatory role of NO in the modulation of adhesion molecules expression. In particular, they showed in IL-1α-stimulated endothelial cells that NO reduces VCAM-1 expression by 35–55% and influences, though to a lesser extent, E-selectin and ICAM-1 [79], thus suggesting both a direct and an indirect role of NO in the regulation of vascular reactivity.

**Figure 3:**
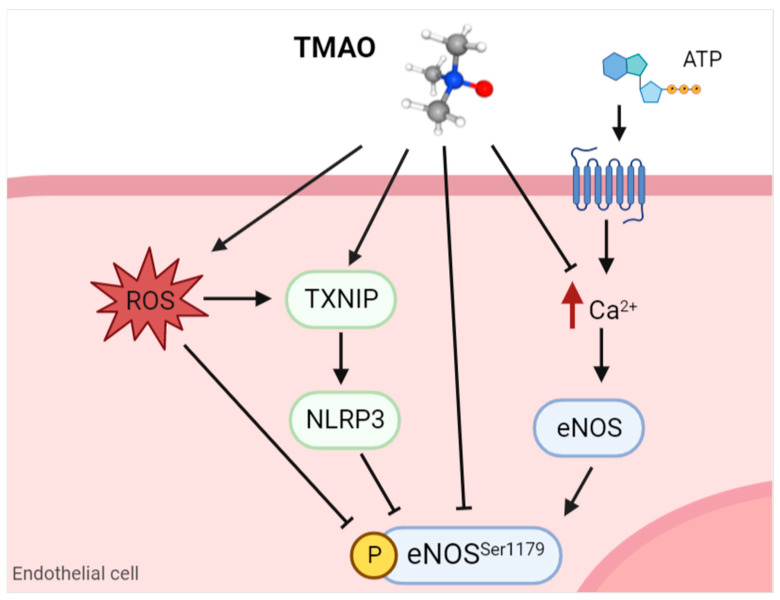
Schematic representation of how TMAO can reduce eNOS activity in endothelial cells. TMAO can directly inhibit eNOS phosphorylation or it can indirectly modulate its activity through the increase of ROS, activation of NLRP3, or decrease in intracellular calcium in purinergic response. TMAO: trimethylamine N-oxide; ATP: adenosine triphosphate; ROS: reactive oxygen species; TXNIP: thioredoxin-interactive protein; NLRP3: nucleotide-binding domain, leucine-rich-containing family, pyrin domain-containing-3; eNOS: endothelial nitric oxide synthase. Image created with Biorender.com.

Different studies focus on the involvement of TMAO in the impairment of NO release, schematically represented in Figure 3. In particular, TMAO seems to reduce NO synthesis by eNOS, both in human umbilical vein endothelial cells (HUVECs) and bovine aortic endothelial cells (BAE-1) [73,74], though through different mechanisms. Indeed, in the first cellular model, TMAO inhibits eNOS and NO release through oxidative stress and thioredoxin-interactive protein (TXNIP)-nucleotide-binding domain, leucine-rich-containing family, and pyrin domain-containing-3 (NLRP3) inflammasome activation [74], while in BAE-1 cells, TMAO affects purinergic-induced intracellular calcium increase, eNOS phosphorylation, and NO release [73]. Furthermore, in vivo studies support these results, showing that TMAO chronic supplementation in C57BL/6N mice induces eNOS impairment, causing a decreased NO release in carotid arteries [69]. On the contrary, Jomard and collaborators found that TMAO does not modify eNOS phosphorylation and NO release either in human aortic endothelial cells (HAECs) or rat aortas, suggesting no relation between TMAO and endothelial dysfunction [71]. The discussed in vitro studies suggest controversial results on the effect of TMAO in inducing endothelial dysfunction, and these could be ascribed to different cellular models (HUVECs vs. BAE-1 vs. HAECs), time, and different treatment concentrations. Indeed, reduced eNOS expression at the mRNA and protein level, and thus NO release, were assessed in HUVECs only at higher treatment concentrations of TMAO (300µM [74]), very far from the physiological plasma concentrations assessed in healthy subjects (from 3 µM to 10 µM) [80]. While no effect on eNOS protein expression was detected, a reduction in its phosphorylation and NO release was detectable in BAE-1 cells only after 24 h of treatment at a relatively high concentration (100 µM) compared to physiological ones [73]. Finally, TMAO treatment for 2 h at a pharmacological concentration of 10^−4^ M induced a reduction in eNOS phosphorylation but did not change NO synthesis in HAECs [71]. Upon analysis of these different in vitro findings, it can be deduced that TMAO may play a role in modulating NO release, although the pathway involved remains to be clearly elucidated.

### 3.2. Adhesion Molecules

Activated endothelial cells express different adhesion molecules that are directly involved in the induction of endothelial dysfunction and subsequent atherosclerosis. It is now widely accepted that vascular damage is defined by several steps, each identified by the expression of specific molecules on the plasma membrane. The first adhesion molecules involved are P- and E-selectins, and vascular cell adhesion molecule-1 (VCAM-1) [26,81]. While selectins slow down leukocyte flow through binding to their carbohydrates ligands, VCAM-1 has been recognized to be gradually expressed in the early stages of atherosclerotic lesions and its function is to recruit and strongly bind to monocytes [82]. Among the adhesion molecules widely expressed by endothelial cells in late atherosclerotic lesions, there is intercellular adhesion molecule-1 (ICAM-1), which blocks and allows leukocyte transmigration out of the blood vessel. ICAM-1 activation is mediated by pro-inflammatory cytokines and enhances inflammatory responses in a positive feedback, exacerbating the atherosclerotic burden [83].

Seldin and collaborators show that primary HAECs treated with TMAO express higher levels of ICAM-1 and E-selectin, prompting TMAO as an inducer of adhesion molecules involved in the late endothelial dysfunction [75]. These same results were obtained in a recent study by Saaoud et al. that demonstrate an increase in mRNA levels of ICAM-1 after TMAO treatment of HAECs [48]. Even though the cells were treated with high concentrations of the molecule (200 and 600 µM, respectively) in both these studies, the final results do not show any functional activation of the adhesion molecule after exposure to TMAO, so these data need to be interpreted with caution when translated into a clinical environment. Furthermore, TMAO induced increased levels of ICAM-1, both at the protein and mRNA level, in the aortic arch of 8-week-old C57BL/6J mice supplemented with 1 mmol/L of TMAO [77]. Ma and collaborators demonstrated that TMAO could modulate the expression of some adhesion molecules in the development of atherosclerosis. Indeed, they showed that TMAO induces the activation of NF-kB and the subsequent expression of VCAM-1, both in HUVECs and in mice aorta endothelial cells. Furthermore, the authors do not see changes in the expression of ICAM-1 after TMAO treatment, suggesting that the molecule is involved in the early stages of atherosclerotic burden [76].

### 3.3. IL-6

The cytokine IL-6 has shown dual behavior towards the activation of pro-inflammatory and anti-inflammatory responses [84]. Concerning the cardiovascular system and the role of IL-6 in atherosclerosis development, different studies have demonstrated its possible damaging effect in modulating vascular smooth muscle cell (VSMC) proliferation, endothelial cells, and platelet activation [85]. Moreover, high levels of IL-6 have been detected in atherosclerotic lesions, suggesting a possible implication of the molecule in the induction of the pro-inflammatory response [86].

Variations in the synthesis of IL-6 after TMAO treatment have been highlighted by Seldin and collaborators, who showed higher expression of IL-6 at the mRNA level both in aortas from LDL^-/-^ female C57BL/6J mice injected with TMAO and in cultured HAECs and VSMCs treated with the molecule [75]. For this set of experiments as well, the authors used a high concentration of TMAO (200µM for 6 h) and they did not investigate protein levels of IL-6, so the direct role of TMAO in the modulation of this angiocrine factor has to be evaluated taking these aspects into consideration. Moreover, the same results were obtained in the aortic arch of ApoE^-/-^ male C57BL/6J mice in which a higher expression of IL-6 was detected after TMAO supplementation [77]. Finally, a recent work by Zhou et al. confirmed the results in cell culture, showing that IL-6 expression is increased in HUVECs and VSMCs after TMAO treatment [78]. In this work, the authors suggest that treatment with 1000 µM TMAO for 24 h enhances the expression of IL-6 both at the mRNA and protein level, and that this pro-inflammatory effect of TMAO is preceded by an increase in reactive oxygen species that would appear to be the first response following cell treatment with the molecule.

Even if more in-depth research is needed to confirm the pro-inflammatory response to TMAO, all the data presented suggest a possible involvement of the molecule triggering an inflammatory response through an increase in IL-6 expression.

## 4. TMAO Modulation of Angiocrine Factors Involved in Other Pathological Onsets

Previous paragraphs present recent findings which describe TMAO as a direct modulator of some angiocrine factors involved in the onset of endothelial dysfunction. This new section presents studies in which TMAO regulates the activity of further angiocrine factors that take part in different pathological conditions other than endothelial dysfunction.

Among all, TMAO shows a direct modulation of VEGF-A, a pro-atherogenic factor, according to Camaré and collaborators [87]. In this regard, two different studies warrant discussion in this review: the first work showed an inverse relation between TMAO and VEGF-A; indeed, a reduction in TMAO plasma concentrations was associated with an increase in VEGF-A in obese patients, suggesting a protective role of the diet-derived molecule [88]. In the second study, concentrations of TMAO up to 250 µM stimulate VEGF-A secretion by HCT-116, a colorectal cancer cell model, and seem to promote tumor angiogenesis [89].

Moreover, TMAO seems to also be involved in the modulation of transforming growth factor (TGF)-β. In particular, treatment with 300 µM TMAO increased the expression of TGF-β receptor type 1, thus aggravating fibrosis, in an in vivo model of myocardial infarction and in isolated neonatal cardiac mouse fibroblasts [90]. Furthermore, TMAO levels were higher in chronic kidney disease patients who also showed increased circulating TGF-β levels, directly involved in the development of renal fibrosis [91].

Finally, we reviewed the literature regarding TMAO and IL-8. Indeed, in rats with myocardial infarction-induced heart failure, elevated plasma levels of TMAO and IL-8, as well as higher expression of IL-8 receptors, have been observed [92]. Results obtained from a cross-sectional study in African American adults at risk of cardiovascular disease showed that TMAO induces hyperlipidemia through proprotein convertase subtilisin/kexin type 9 (PCSK9) and this pathway is mediated by IL-8 [93]. Lastly, Macpherson and collaborators showed that TMAO is associated with high circulating levels of IL-8 and other pro-inflammatory cytokines in patients with common variable immunodeficiency (CVID) and presented TMAO as a possible trigger for pathological outcome [94].

## 5. Conclusions

The present work aimed to present the role of TMAO, a diet-derived compound, in the modulation of some angiocrine factors related to atherosclerosis development. Indeed, recent evidence underlines that patients with endothelial dysfunction and atherosclerosis have higher levels of plasmatic TMAO [63], but its role as a direct cause or a marker of pathology is still controversial. Different angiocrine factors, already recognized to play a pivotal role in the development of endothelial dysfunction and its related comorbidities, have been considered in this work, with a special focus on those that seem to be directly regulated by TMAO. The presented results regarding only the last actors of intracellular pathways could be explained by the direct modulation of NF-kB by TMAO [95].

In light of the presented data about TMAO regulation of angiocrine factors involved in the development of endothelial dysfunction, the role of the molecule still seems controversial and further in vitro and in vivo studies are needed to characterize it definitively as a cardiovascular marker or a risk factor.

## Figures and Tables

**Table 1:** TMAO direct modulation of some angiocrine factors.

Angiocrine Factor	Modulation Effect by TMAO	References
NO	Reduction in eNOS phosphorylation and NO release in HUVECs and BAE-1 cells	[73,74]
	Reduction in eNOS phosphorylation and NO release in the carotid artery of C57BL/6N mice	[69]
	No variation in eNOS phosphorylation and NO release in HAECs and rat aortas	[71]
Adhesion molecules	Enhancement of ICAM-1 and E-selectin expression in HAECs	[48,75]
	Induction of VCAM-1 expression in HUVECs and mice aortic endothelial cells	[76]
IL-6	Enhanced expression at the mRNA level in aortas from C57BL/6J mice and cultured HEACs and VSMCs	[75]
	Increased expression at the mRNA level in the aortic arch of C57BL/6J mice	[77]
	Increased expression at mRNA and protein level in HUVECs and VSMCs	[78]

## Data Availability

Not applicable.
